# Regional seropositivity for *Borrelia burgdorferi* and associated risk factors: findings from the Rhineland Study, Germany

**DOI:** 10.1186/s13071-022-05354-z

**Published:** 2022-07-04

**Authors:** Annabell Coors, Max J. Hassenstein, Gérard Krause, Tobias Kerrinnes, Manuela Harries, Monique M. B. Breteler, Stefanie Castell

**Affiliations:** 1 German Center for Neurodegenerative Diseases (DZNE), Population Health Sciences, Venusberg-Campus 1/99, 53127 Bonn, Germany; 2 Department of Epidemiology, Helmholtz Centre for Infection Research (HZI), Braunschweig, Germany; 3 PhD Programme “Epidemiology”, Braunschweig-Hannover, Germany; 4 German Center for Infection Research (DZIF), Braunschweig, Germany; 5 Hannover Medical School (MHH), Hannover, Germany; 6 TWINCORE, Centre for Experimental and Clinical Infection Research, a Joint Venture of the Hannover Medical School, Helmholtz Centre for Infection Research, Hannover, Germany; 7 Department of RNA-Biology of Bacterial Infections, Helmholtz Institute for RNA-Based Infection Research, Würzburg, Germany; 8 Institute for Medical Biometry, Informatics and Epidemiology (IMBIE), Faculty of Medicine, University of Bonn, Bonn, Germany

**Keywords:** Lyme disease, Seroepidemiological studies, Immunoglobulin G, Immunoglobulin M, Antibodies, Ticks, Enzyme-linked immunosorbent assay, Immunoblotting, Tick-borne diseases, Germany

## Abstract

**Background:**

Lyme borreliosis is the most prevalent vector-borne disease in Europe, and numbers might increase due to climate change. However, borreliosis is not notifiable in North Rhine-Westphalia (NRW), Germany. Hence, little is known about the current human seroprevalence in NRW. However, the proportion of *Borrelia burgdorferi* sensu lato-infected ticks has increased in a NRW nature reserve. The literature suggests increasing age and male sex as risk factors for seropositivity, whereas the influence of socioeconomic status is controversial. Thus, we aimed to determine regional seropositivity for *Borrelia burgdorferi* sensu lato (*B. burgdorferi* s.l.) and its risk factors in the Rhineland Study population in Bonn, NRW, and to compare it with previous surveys to evaluate potential effects of climate change.

**Methods:**

We assessed seropositivity in 2865 Rhineland Study participants by determining immunoglobulin G (IgG) and immunoglobulin M (IgM) antibodies for *B. burgdorferi* s.l. using a two-step algorithm combining enzyme-linked immunosorbent assay tests and line immunoblots. We calculated the odds of being classified as IgG or IgM positive as a function of age, sex, and educational level using binomial logistic regression models. We applied varying seropositivity classifications and weights considering age, sex and education to compensate for differences between the sample and regional population characteristics.

**Results:**

IgG antibodies for *B. burgdorferi* s.l. were present in 2.4% and IgM antibodies in 0.6% of the participants (weighted: 2.2% [IgG], 0.6% [IgM]). The likelihood of IgG seropositivity increased by 3.0% (95% confidence interval [CI] 1.5–5.2%) per 1 year increase in age. Men had 1.65 times the odds for IgG seropositivity as women (95% CI 1.01–2.73), and highly educated participants had 1.83 times the odds (95% CI 1.10–3.14) as participants with an intermediate level of education. We found no statistically significant link between age, sex, or education and IgM seropositivity. Our weighted and age-standardized IgG seroprevalence was comparable to the preceding serosurvey German Health Interview and Examination Survey for Adults (DEGS) for NRW.

**Conclusions:**

We confirmed that increasing age and male sex are associated with increased odds for IgG seropositivity and provide evidence for increased seropositivity in the highly educated group. *B. burgdorferi* s.l. seropositivity remained constant over the past decade in this regional German population.

**Graphical abstract:**

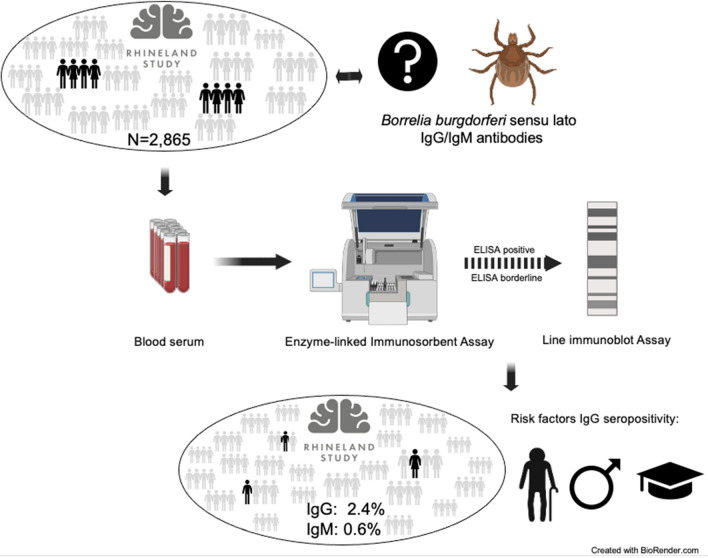

## Background

Lyme borreliosis is the most prevalent tick-borne disease in Europe [1]. From 2013 to 2017, yearly reported incidence for Lyme borreliosis in German states with disease notification ranged from 26 to 41 reported cases per 100,000 inhabitants [2]. However, incidence data from German health insurance funds for 2019 indicate 429 diagnoses per 100,000 insured persons for Germany [3]. For North Rhine-Westphalia (NRW), the insurer Nordrhein reported 99 diagnoses per 100,000 insured persons and the insurer Westfalen-Lippe reported 135 diagnoses per 100,000 insured persons [3]. In addition, yearly incidence between certain regions varies enormously; for example, in 2017 and within Mecklenburg-Western Pomerania, over 160 cases per 100,000 persons were reported in the west, and less than 40 cases per 100,000 persons were reported in the east. Moreover, changes over time have also been observed recently; Lyme borreliosis incidence in Bavaria, for instance, increased from 23.2 per 100,000 inhabitants in 2015 to 47.4 per 100,000 inhabitants in 2020 [4]. In all, the picture is highly heterogeneous regarding information from different data sources, regions and time.

Spirochaetes of the genospecies complex *Borrelia burgdorferi* sensu lato (*B. burgdorferi* s.l.), the causal agent of Lyme borreliosis [5], are detectable in about 3–35% of ticks in Germany [6–12]. Of at least 18 unique known genospecies of *B. burgdorferi* s.l., *B. afzelii*, *B. burgdorferi* and *B. garinii* are the three major genospecies in Europe [13], transmitted to humans by ticks (*Ixodes ricinus*) [14]. Ticks are only active if the weekly mean temperature exceeds 7 °C [15]. Additionally, the time people spend outdoors, i.e. potential tick exposure, generally increases with rising air temperature, except for poor or extreme weather conditions [16]. Therefore, reported infections with the *Borrelia* complex occur in Europe mainly between March and October, peaking from June to August [2]. Since climate change is leading to higher mean temperatures [17, 18], tick season will potentially be prolonged [19] and could continue throughout the winter in Germany if temperatures are mild [20]. An increase in annual air temperature by 1 °C was observed for NRW (1961–1990 compared to 1991–2020) [21]. At the same time, the number of days with ice and frost decreased. The warmer winters with less snow may promote an earlier food supply for wild boars and other potential hosts for ticks (*Ixodes ricinus*) [22], and may potentially increase tick activity throughout the winter season [20]. Thus, climate change is evident in our region of interest, and its potential consequences should be investigated. However, since there is a complex interplay between environmental factors (e.g. climate), ticks, available hosts, *Borrelia* genospecies distributions and anthropogenic factors (e.g. recreational activities) [23], it is difficult to predict whether or to what extent climate change will change the infection risk with *B. burgdorferi* s.l. [19, 24, 25]. Tick density was found to increase in a nature reserve, Siebengebirge, near Bonn, from 1987/1989 until 2008 [26]. Also, rising tick infection proportions have been reported for this area, where the number of ticks carrying spirochaetes increased 2.5-fold within a decade [27]. However, the *Borrelia* genospecies distribution also changed over this time period. In 2001, *B. valaisiana*, a non-pathogenic species for humans [28], was the dominant species, whereas in 2007 *B. garinii* and *B. afzelii* were the dominant species in the Siebengebirge. Thus, the distribution of *Borrelia* genospecies has to be taken into account when looking at changes in tick infection proportions. Further, there were considerable differences in *Borrelia* genospecies distributions among the three different tick collection sites in the nature reserve. In other areas such as Hannover, more ticks were found to carry *B. burgdorferi* s.l. than in the Siebengebirge. However, here, the tick infection proportion was constant over a decade, and *B. valaisiana* accounted for only a small proportion of infected ticks during the measurement times [9]. Hence, the current and future development of case numbers may vary considerably between regions, rendering area-specific monitoring necessary, especially since there exists no vaccine against Lyme borreliosis [5].

Lyme borreliosis is notifiable in nine of 16 German states, excluding NRW. For regions without notification systems, serosurveys can provide area-specific estimates on infection risk. Here, the presence of immunoglobulin M (IgM) and immunoglobulin G (IgG) antibodies points towards an infection with Lyme borreliosis, with IgM antibody levels generally detectable sooner and dropping more frequently after a shorter period of time than IgG antibodies [29–31]. One study with 79 Lyme disease patients reported that 10 to 20 years after infection, IgM antibodies still persisted in 10% of the patients with early Lyme disease and 15% of the patients with Lyme arthritis, whereas 25% of the patients with early Lyme disease and 62% of the patients with Lyme arthritis still had IgG antibodies [31]. The most recent serosurvey for *B. burgdorferi* antibodies in Germany was conducted in the study population of the German Health Interview and Examination Survey for Adults (DEGS) from 2008 to 2011 [30, 32]. Out of 6965 adults aged between 18 and 79 years, 9.4% were classified as seropositive [32]. The reported seroprevalence for NRW was 5.1% (95% confidence interval [CI] 3.8–6.3%) for the years 1997–1999 (German National Health Interview and Examination Survey 1998, BGS98) and 5.3% (95% CI 3.7–7.0%) for the years 2008 to 2011 (DEGS) [30]. However, the serosurvey was limited to IgG antibodies, and seropositivity may have changed in the past decade. Thus, updated numbers on seropositivity are necessary to detect potential trends in infection risk.

Further, studies that use a two-step antibody testing procedure may differ from current guidelines, for example, MIQ 12 [33]. According to the MIQ 12 guideline, the final result for IgG or IgM serostatus is classified as seropositive in the case of a positive or borderline enzyme-linked immunosorbent assay (ELISA) and a subsequent positive immunoblot. Woudenberg et al. [30] and Wilking et al. [32] used a more sensitive classification for their serosurvey results, as they additionally classified borderline line blot results with prior positive ELISA as positive. On the contrary, Kalmár et al. [34] applied a classification of seropositive samples corresponding to MIQ 12. Moreover, some studies have additionally reported serological results that have been corrected for sample deviations from the general population [32]. These differences in classification and testing procedures, such as utilizing different test kits, make it difficult to compare the results between studies [30, 32, 34].

Besides known age effects on seropositivity [32], higher seropositivity in men than women has been reported [32], although analyses of clinical cases, such as on the basis of disease notification data, report higher incidence in women [2]. Other potential risk factors remain under discussion, namely the role of socioeconomic status (SES) [30, 35–39].

Since the risk of acquiring Lyme borreliosis may be increasing as a consequence of a complex interplay between climate change, anthropogenic factors and number of infected ticks, the primary aim of our investigation was to obtain current estimates of IgG and IgM seropositivity for *B. burgdorferi* s.l. in the Rhineland Study population in Bonn, Germany, and to compare them to previous estimates from other studies with data from NRW, the federal state in which Bonn is situated. Secondly, we assessed risk factors for seropositivity including the controversially discussed SES. Lastly, we investigated both IgG and IgM for a full picture on *Borrelia* serology, and applied different algorithms for classification of samples as positive to assess the extent to which this resulted in differences in seropositivity and its risk factors and to achieve comparability between studies to assess potential effects of climate change [17].

## Methods

We used data from 2888 participants of the Rhineland Study who provided blood samples between February 2018 and February 2020. The Rhineland Study is a community-based cohort study in Bonn, Germany. All inhabitants of two geographically defined areas in Bonn who are at least 30 years old and have sufficient command of the German language to provide written informed consent can participate upon invitation. Eligibility is irrespective of health status. We did not offer any financial incentives for participation. Participants of the Rhineland Study underwent 8 h of examinations. We obtained sociodemographic information on age, sex and education using standardized interviews and questionnaires.

A DIN EN ISO [German Institute for Standardization/European standard/International Organization for Standardization] 15189-accredited medical laboratory conducted the antibody analysis on the sera with a two-step algorithm according to MIQ 12 (microbiologic-infectiologic quality standard) [33]. The commissioned laboratory is ISO 9001-certified with expertise in the medical-serological routine diagnostics of Lyme borreliosis. Firstly, the sero-samples underwent ELISA to test for IgG and IgM antibodies with > 99% sensitivity for both IgG and IgM and 97% specificity for IgG and 98.8% for IgM [*B. afzelii* + VlsE IgG Europe ELISA, and *B. afzelii* IgM ELISA; Virotech Diagnostics GmbH]. The antigens used in the ELISA are a mixture of the *B. afzelii* strain Pko, the *B. garinii* strain PBr and the *B. burgdorferi* strain ZS7. The ELISA testing procedure was fully automated [DSX^®^ ELISA Processors; Dynex Technologies]. Secondly, the presence of detected antibodies in positive and borderline samples was verified [ProfiBlot^®^ Automated Systems; Dynex Technologies] by line immunoblots with a test sensitivity of > 99.9% and specificity of 98% [WE225 Borrelia Europe plus TpN17 LINE IgG and WE224 Borrelia Europe LINE IgM; Virotech Diagnostics GmbH]. The line immunoblots considered the antigens OpsC (p23) from *B. afzelii*, VlsE recombinant from *B. burgdorferi* B31, p39 (BmpA) recombinant from *B. afzelii* PKo, DbpA (Pko) and DbpA (PBi, PBr, A14 S) from *B. bavariensis* PBi and *B. garinii* PBr, p58 (OppA-2) recombinant from *B. bavariensis* PBi, p83/100 recombinant from *B. afzelii* PKo, and EBV VCA-gp125 (affinity-purified) for exclusion diagnostics. The classification of IgG and IgM antibodies considered Virotech units (VU), an arbitrary antibody quantification scale for ELISA, as follows: negative: VU < 9, borderline: VU >  = 9 and VU <  = 11, positive: VU > 11. Finally, all generated data were imported to LabImage^®^ LA Software [Kapelan Bio-Imaging Solutions] to detect, combine and interpret the immunoblots. A medical-technical assistant conducted the technical verification, and a medical doctor for microbiology, virology and infectious disease epidemiology validated the results. In our primary analysis, we classified all subjects with either positive or borderline ELISA and subsequent positive line immunoblot as seropositive and all other combinations as non-seropositive. We performed two binomial logistic regression models to calculate the odds of being classified as IgG-positive or IgM-positive as a function of age, sex and educational level.

We classified educational level according to the International Standard Classification of Education (ISCED) 2011 as low (completed lower secondary education or below), middle (completed upper secondary education up to completed bachelor’s degree or equivalent) and high (completed master’s degree or equivalent up to completed doctoral or equivalent) as a proxy for SES.

In sensitivity analyses, we investigated whether age, sex and educational level were associated with IgG and IgM VU, the original ELISA quantification scales without modification and regardless of serostatus, running two linear regression models with age, sex and education as predictors. For this analysis, we present unstandardized regression coefficients. Furthermore, we ran two ordinal logistic regression models with negative, borderline and positive IgG and IgM serostatus as ordinal outcome and age, sex and education as predictors.

Finally, to compare our seroprevalence results with previous studies from approximately the same region, such as the DEGS serosurvey [30], we applied an alternative seropositivity classification and classified the following combinations as seropositive: positive ELISA and positive or borderline immunoblot, borderline ELISA and positive immunoblot [30]. We additionally report the seropositivity results based on ELISA only. Furthermore, to correct for sample deviations from the general population in NRW, we also calibrated the crude seroprevalence results with regard to age and sex distributions that were reported in the latest census in 2011 for the individuals aged 30 years and above from NRW, and with regard to the distribution of educational status that was reported in the last micro-census survey in 2020 for the total German population (www.destatis.de [40]). To compare our seroprevalence with the seroprevalence reported for BGS98 and DEGS [30], we weighted our study population by sex and education as stated above, and age-standardized by the respective age distribution of the studies we compared our results with [41, 42].

We carried out all analyses in RStudio (version 1.3.959, R-base version 4.0.3). We used the glm function of the stats package with a logit-link for the logistic regression models and the lm function of the stats package for the linear regression models [43]. For the ordinal regression, we utilized the polr() function within the MASS package [44]. The variance inflation factor (VIF), as test for multicollinearity, remained below 2 for all models (using the car package) [45]. To calculate the calibration weights, we used the survey package [46] and trimmed the weights to fit into the interval of 0.3 to 3.

## Results

Of the 2888 participants, serostatus could be determined for all but one participant, and information on educational level was available for all but 22 participants, resulting in 2865 participants for analysis (Table 1). The participants’ median age was 55 years (interquartile range: 45 to 65 years, min–max: 30 to 94 years), 55.8% were women, and 52.6% had high, 45.7% medium and 1.7% low educational levels, respectively.

**Table 1:** Population characteristics and stratified crude seroprevalence of IgG/IgM antibodies for *B. burgdorferi* s.l. and odds ratios from logistic regression analysis

Characteristics	IgG seropositive/total	IgG seroprevalence [%]	IgM seropositive/total	IgM seroprevalence [%]	IgG: odds ratios (95% CI), *P*-value	IgM: odds ratios (95% CI), *P*-value
All subjects	69/2865	2.4	18/2865	0.6	**–**	–
Age (years)	–	–	–	–	**1.03 (1.02–1.05), < 0.001**	0.99 (0.96–1.03), 0.748
Sex						
Women	28/1600	1.8	11/1600	0.7	Ref	Ref
Men	41/1265	3.2	7/1265	0.6	**1.65 (1.01–2.73), 0.049**	0.79 (0.29–2.02), 0.627
Education (ISCED 2011)						
Low	1/50	2.0	0/50	0.0	1.10 (0.06–5.45), 0.928	0.00 (NA), 0.988
Middle	23/1309	1.8	8/1309	0.6	Ref	Ref
High	45/1506	3.0	10/1506	0.7	**1.83 (1.10–3.14), 0.023**	1.09 (0.42–2.92), 0.853

Educational level was determined using the International Standard Classification of Education 2011 (ISCED) and was coded as low (lower secondary education or below), middle (upper secondary education to undergraduate university level) and high (postgraduate university study). The two columns on the right display the odds of having a positive serostatus for either IgG or IgM antibodies as a function of age, sex and education. Seropositive serostatus refers to all subjects that had a positive or borderline ELISA and a subsequent positive line immunoblot (MIQ 12 algorithm). Associations with a *P*-value below 0.05 are shown in bold

*IgG* immunoglobulin G antibodies; *IgM* immunoglobulin M antibodies; *Ref* reference group in the logistic regression model; *CI* confidence interval; *NA* not applicable because there were no participants in the low-education IgM seropositivity group

**Fig. 1:**
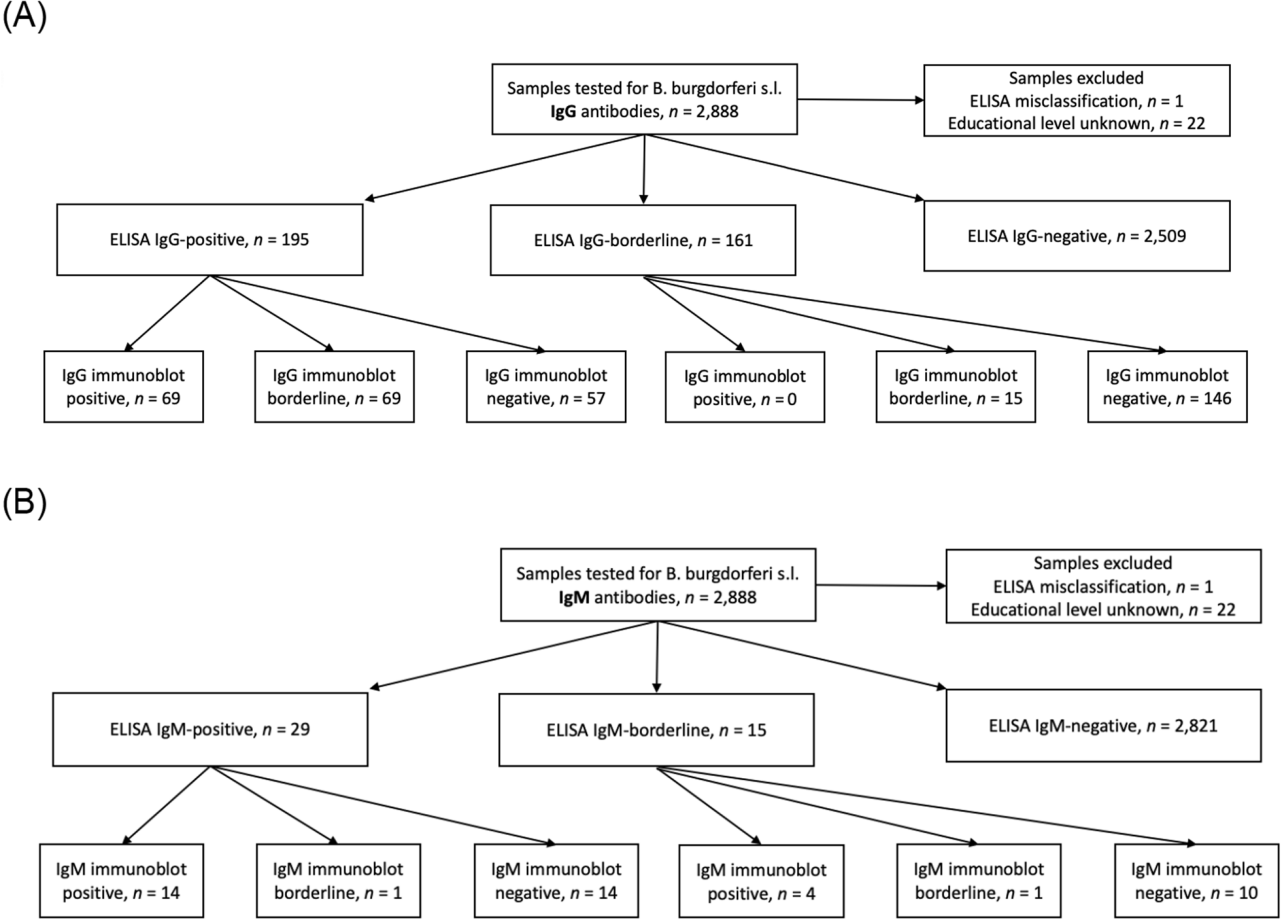
Results for ELISA and line immunoblot. **A** Flowchart for IgG antibodies; **B** Flowchart for IgM antibodies

Figure 1 presents the flowcharts from our IgG/IgM testing procedure, including the test results from ELISA and line immunoblot.

Table 2 presents the crude, weighted and additionally age-standardized proportions for seropositivity for our study sample, differentiated by varying classifications. Based on the ELISA results with immunoblot confirmation, 2.4% (*n* = 69) were classified as IgG-positive, 0.6% (*n* = 18) as IgM-positive and 0.1% (*n* = 3) as both IgG- and IgM-positive. Thus, we detected antibodies for *B. burgdorferi* s.l. in 2.9% of the participants. The relative frequencies for IgG seropositivity were higher in men than in women (3.2% of men versus 1.8% of women) and frequencies were comparable between the sexes for IgM seropositivity (0.7% of women and 0.6% of men). When considering the relative frequencies of ELISA-seropositive samples without immunoblot confirmation, seropositivity was higher for both IgG (6.8% in ELISA-only classification versus 2.4%) and IgM (1.0% versus 0.6%). When we applied the algorithm used in DEGS and BGS98 [30], 4.8% were IgG-positive, 0.7% were IgM-positive and 0.2% were both IgG- and IgM-positive (crude data). The weighted seropositivity results with the more stringent classification algorithm for IgG were 2.2% (95% CI: 1.6–2.7%) compared to 2.4% in the crude analysis. The weighted seropositivity result for IgM resulted in the same point estimate of 0.6% (95% CI: 0.3–0.9%). When we applied the algorithm used in the DEGS and BGS98 serosurvey and additionally age-standardized our weighted sample to make it comparable to the DEGS and BGS98 populations, respectively, the estimated IgG proportions were 4.7% (95% CI: 3.9–5.4%, DEGS age standardization) and 4.2% (95% CI: 3.4–4.9%, BGS98 age standardization) compared to 5.3% (95% CI 3.68–6.97, DEGS) and 5.1% (95% CI 3.83–6.28 BGS98) previously estimated for NRW.

**Table 2:** Crude, weighted, and weighted and age-standardized seroprevalence based on varying seropositivity classification algorithms

Seropositivity combinations	Crude proportion (%), *n* = 2865	Weighted proportion (%) with 95% CI, *n* = 2858	Weighted & age-standardized (BGS98) proportion (%) with 95% CI, *n* = 2858	Weighted & age-standardized (DEGS) proportion (%) with 95% CI, *n* = 2858
IgG seropositivity				
IgG positivity–ELISA borderline or positive & positive immunoblot^a^	2.4	2.2 (1.6, 2.7)	1.8 (1.4, 2.3)	2.2 (1.6, 2.7)
IgG positivity–positive ELISA	6.8	6.4 (5.5, 7.3)	6.0 (5.1, 6.9)	6.5 (5.6, 7.4)
IgG positivity–positive ELISA & borderline or positive immunoblot or borderline ELISA & positive immunoblot^b^	4.8	4.6 (3.8, 5.3)	4.2 (3.4, 4.9)	4.7 (3.9, 5.4)
IgM seropositivity				
IgM positivity–ELISA borderline or positive & positive immunoblot^a^	0.6	0.6 (0.3, 0.9)	0.6 (0.3, 0.8)	0.6 (0.3, 0.8)
IgM positivity–positive ELISA	1.0	1.0 (0.6, 1.3)	0.9 (0.5, 1.2)	1.0 (0.6, 1.3)
IgM positivity–positive ELISA & borderline or positive immunoblot or borderline ELISA & positive immunoblot^b^	0.7	0.6 (0.3, 0.9)	0.6 (0.3, 0.9)	0.6 (0.4, 0.9)
IgG and IgM seropositivity combined				
IgG positivity & IgM positivity–ELISA borderline or positive & positive immunoblot^a^	0.1	< 0.1 (0.0, 0.2)	0.1 (0.0, 0.3)	0.1 (0, 0.2)
IgG positivity & IgM positivity–positive ELISA	0.4	0.5 (0.2, 0.7)	0.4 (0.2, 0.7)	0.5 (0.2, 0.8)
IgG positivity & IgM positivity–positive ELISA & borderline or positive immunoblot or borderline ELISA & positive immunoblot^b^	0.2	0.3 (0.1, 0.5)	0.3 (0.1, 0.5)	0.3 (0.1, 0.5)

To correct for differences between the sample and the German population regarding population characteristics, we applied weights considering age, sex (census 2011, www.destatis.de) and education (micro-census 2020, www.destatis.de) [40]. To make our proportions comparable to those numbers reported in previous studies for the DEGS and BGS98 cohorts, we applied weights considering sex and education and then age-standardized the weighted proportions to reflect the age distribution of the DEGS and BGS98 cohorts.

^a^Corresponding to the MIQ 12 standard [33]

^b^Classification used in Woudenberg et al. [30]

*N* number of participants; *M* mean; *IgG* immunoglobulin G antibodies; *IgM* immunoglobulin M antibodies; *SD* standard deviation; *IQR* interquartile range

The median of the VU for the entire sample was 4.3 (interquartile range (IQR): 4.1) for IgG and 1.6 (IQR:1.9) for IgM. For the seropositive participants, the median was 29.1 (IQR: 14.56) for IgG and 13.85 for IgM (IQR: 6.6).

The odds of being classified as IgG-positive increased by 3% (95% CI 1.5–5.2%) per 1 year increase in age (Table 1). In addition, men were 1.65 (95% CI 1.01–2.73) times as likely to be classified IgG-positive as women. Further, a high level of education was associated with 1.83 higher odds (95% CI 1.10–.14) of IgG seropositivity compared with an intermediate level of education. We observed no significantly increased or decreased odds for IgG and IgM seropositivity in the low education group compared with intermediate education. Regarding IgM serostatus, age, sex and educational level were not significantly associated with seropositivity.

When considering VU instead of qualitative classification, IgG units increased and IgM units decreased by 0.02 units per 1 year increase in age (95% CI for IgG units: 0.01–0.03; IgM units: −0.02 to −0.01) (Table 3). Men had on average 0.35 lower IgM units than women (95% CI −0.52 to −0.18), but did not differ statistically significantly from women in IgG units. Educational level was not significantly associated with IgG and IgM units. In our ordinal regression for negative, borderline or positive IgG serostatus (Table 4), we found that men had 1.58 times the odds for borderline or positive versus negative serostatus compared to women (95% CI 1.13–2.22). Also, 1 year higher age was associated with higher odds for borderline or positive versus negative serostatus (95% CI 1.01–1.04). The ordinal model for IgM serostatus (Table 4) yielded no significant results. In both ordinal models, we found no association between education and serostatus. When we reclassified all samples with either a positive ELISA and positive or borderline immunoblot, or a borderline ELISA and positive immunoblot as seropositive according to Woudenberg et al. [30], we found that subjects had 1.02 times the odds of being classified as IgG-positive per 1 year increase in age (95% CI 1.01–1.04) (Table 5). Further, men were 1.82 times as likely (95% CI 1.28–2.60) to be classified as IgG-positive as women, and participants with high or low educational levels did not have increased odds of IgG seropositivity compared with intermediate educational level. We found no significant odds ratios between age, sex or education and IgM seropositivity.

**Table 3:** Associations between age, sex, education and IgG-/IgM virotech units

Outcome	Independent variables	b (95% CI)	*P*-value
IgG in Virotech units	Age	**0.02 (0.01 to 0.03)**	**0.005**
	Sex (ref: women)	0.38 (−0.03 to 0.78)	0.066
	Education high (ref: middle)	0.40 (−0.01 to 0.81)	0.056
	Education low (ref: middle)	−0.18 (−1.71 to 1.35)	0.820
IgM in Virotech units	Age	**−0.02 (−0.02 to −0.01)**	**< 0.001**
	Sex (ref: women)	**−0.35 (−0.52 to −0.18)**	**< 0.001**
	Education high (ref: middle)	−0.07 (−0.24 to 0.10)	0.436
	Education low (ref: middle)	−0.29 (−0.92 to 0.34)	0.371

The table displays the change in IgG and IgM Virotech units per 1 year increase in age and the differences between men and women and participants of different educational levels in IgG and IgM Virotech units. Associations with a *P*-value below 0.05 are shown in bold

*Ref* reference; *CI* confidence interval; b = unstandardized regression coefficient; *IgG* immunoglobulin G antibodies; *IgM* immunoglobulin M antibodies

**Table 4:** Odds ratios from ordinal logistic regression for IgG/IgM seropositivity

Outcome	Independent variables	Odds ratios (95% CI)	*P*-value
IgG-seropositive or borderline (ref: negative)	Age	**1.03 (1.01–1.04)**	**< 0.001**
	Sex (ref: women)	**1.58 (1.13–2.22)**	**0.007**
	Education high (ref: middle)	1.38 (0.98–1.95)	0.065
	Education low (ref: middle)	0.42 (0.02–1.97)	0.393
IgM-seropositive or borderline (ref: negative)	Age	0.99 (0.40–2.44)	0.642
	Sex (ref: women)	0.83 (0.83–0.83)	0.688
	Education high (ref: middle)	1.06 (0.15–7.30)	0.907
	Education low (ref: middle)	**0.00 (0.00–0.00)**	**< 0.001**

The table displays the odds of being either IgG/IgM borderline or seropositive vs seronegative while adjusting for age, sex and educational level. Associations with a *P*-value below 0.05 are shown in bold

*Ref* reference; *CI* confidence interval; *IgG* immunoglobulin G antibodies; *IgM* immunoglobulin M antibodies

**Table 5:** Associations between age, sex, education and seropositivity as defined by either positive ELISA and positive or borderline immunoblot or borderline ELISA and positive immunoblot result

Outcome	Independent variables	Odds ratios (95% CI)	*P*-value
IgG seropositive (ref: non-positive)	Age	**1.02 (1.01–1.04)**	**< 0.001**
	Sex (ref: women)	**1.82 (1.28–2.60)**	**0.001**
	Education high (ref: middle)	1.41 (0.99–2.03)	0.063
	Education low (ref: middle)	0.49 (0.03–2.32)	0.486
IgM seropositive (ref: non-positive)	Age	1.00 (0.96–1.03)	0.850
	Sex (ref: women)	0.73 (0.27–1.83)	0.511
	Education high (ref: middle)	0.99 (0.39–2.54)	0.982
	Education low (ref: middle)	0.00 (NA)	0.988

The table displays the odds of having either a positive ELISA result and a subsequent positive or borderline immunoblot or a borderline ELISA result and a subsequent positive immunoblot for either IgG or IgM antibodies as a function of age, sex and education. Associations with a *P*-value below 0.05 are shown in bold

*IgG* immunoglobulin G; *IgM* immunoglobulin M; *ref* reference; *CI* confidence interval; *NA* not applicable because there were no participants in the low-education IgM seropositivity group

## Discussion

The weighted seroprevalence values for antibodies for *B. burgdorferi* s.l., applying a stringent classification scheme, were 2.4% for IgG, 0.6% for IgM, and 0.1% for both IgG and IgM antibodies. We demonstrated that varying classification algorithms found in the literature for seropositivity led to differences in seroprevalence. However, using identical classification as BGS98 and DEGS, our weighted and additionally age-standardized seroprevalence values were within the confidence intervals of the seroprevalence values previously reported for NRW [30]. Hence, we found no evidence for increased IgG antibody seroprevalence for *B. burgdorferi* s.l. in light of climate change, despite reported changes in tick distribution and proportion of infection with *Borrelia* in one region in NRW [26, 27].

For IgG seropositivity, we found a statistically significant impact of age, sex and educational level, while for IgM, we found no statistical link between age, sex or education and seropositivity, the latter possibly due to the small proportion of IgM-positive individuals.

As a use case, this study provides, therefore, valuable and detailed information for a region for which data on ticks are worrying from a public health perspective and data on Lyme borreliosis are largely missing. Further, our results highlight the essential requirement for comparable classifications, as they evidently influence seropositivity results and, therefore, must be identical if seropositivity results are to be compared across studies. Large differences between different ELISA systems have already been demonstrated [47] and emphasize that classification algorithms also strongly influence seroprevalence estimates. Furthermore, we recognize that the choice of serology test kit may impact antibody detection. The studies discussed in our work apply different test kits due to availability, which might influence comparability.

In our main analysis, we confirmed the general effect of age for IgG seropositivity that has been reported in previous studies [32, 48]. IgG seropositivity may increase with age due to cumulative lifetime exposure [32, 48]. In line with previous serosurveys, we also found that men had higher odds of having IgG antibodies than women [32]. This stands in contrast to findings on higher incidence of Lyme borreliosis as a clinical manifestation in women compared to men [2, 49, 50]. One reason may be that men are less likely to go to the doctor [51] and might, therefore, less often be diagnosed with Lyme borreliosis than women. However, men and women did not differ in IgM seropositivity. Since only 0.6% of our sample was IgM-seropositive, it could also be that the statistical power was too low to detect statistically significant effects.

From the IgG unit data, we find that IgG units were on average higher in older participants, which fits to the above-discussed explanation of cumulative lifetime exposure to *B. burgdorferi* s.l. The negative association between age and IgM units could suggest that the IgM-related immune response decreases with age [52]. However, data on the timing of infection would be required to further clarify this finding, as it is possible that younger adults had more recent infections than older adults. An alternative explanation for lower IgM units in older participants may be related to decreased tick exposure, as physical outdoor activity may decline with age [53].

Previous studies discussed a potential interplay between SES, time spent outside (i.e., tick exposure) and *Borrelia* seropositivity [35–39]; in the latest German serosurvey, no influence of SES was found [30]. We are aware of one other study that also used educational level as proxy for SES but grouped it into four different educational levels [37]. This study reported no association between education and seropositivity. However, given that they had a smaller sample size than our study (*N* = 1213), the lower number of participants per category may have limited the statistical power to detect an existing association. We found participants with a higher level of education to have higher odds for IgG seropositivity than individuals with an intermediate level of education. A potential explanation for our finding is differing life circumstances, including the living environment, leisure activities and access to green spaces and nature, resulting in differences in tick exposure [35, 38]. In contrast, we found no significant effect for low educational level compared to intermediate level of education. However, we only had a few persons with a low educational level, and their number may have been too small to find meaningfully interpretable results, supported by a comparably wide confidence interval for the odds ratio. Our sensitivity analyses considering IgG and IgM VU, negative, borderline and positive samples, and the DEGS classification algorithm also found no significant effect for any level of education. Thus, we demonstrate that different classifications affect the detection of risk factors for seropositivity and the seroprevalence.

Our study has limitations. First, we conducted a serosurvey for IgG and IgM antibodies for *B. burgdorferi* s.l., meaning that we did not examine study subjects for clinical disease. Despite high specificity in both the screening and confirmatory tests, we cannot fully exclude the very small chance that cross-reactions may have led to false-positive results among samples, for example, caused by present immune reactions to syphilis [54], relapsing fever [55] or herpes diseases (cytomegalovirus or parvoviruses [56, 57]). Furthermore, our cohort consisted of a local sample from two city districts in Bonn, Germany. Therefore, our study does not represent the whole of NRW; hence, our comparison with the only available previous seroprevalence data of our study region relies on estimates for NRW provided by Woudenberg et al. [30], rendering caution advisable. Furthermore, only subjects aged 30 and older were invited to the study. Thus, we cannot assess the serostatus in the age cohort younger than 30 years, which are potentially prone to exposure during leisure or work activities by design, as discussed elsewhere [32]. In our statistical analysis, we were limited to investigating the risk factors of age, sex and education due to data restrictions and could not include further risk factors for seropositivity evaluated in existing studies, such as migration background, pets within the household or time spent outside [32, 37, 48].

In conclusion, this study provides an important update of IgG antibody seropositivity estimates for *B. burgdorferi* s.l. in Bonn and additionally provides seropositivity estimates for IgM antibodies. Although we hypothesized an increase in seroprevalence due to reports of increased tick density and tick infection proportions in this region over the past few decades [26, 27], we did not detect such increase in seropositivity in our sample compared with the findings from the most recent German serosurveys conducted during 2008–2011 (DEGS) and 1997–1999 (BGS98). We also demonstrated how important the choice of classification is for the comparability of seropositivity results across studies. Spatial variation in tick activity and exposure highlight the need for future studies to investigate IgG and IgM seropositivity in other regions to map trends and enable early action (e.g. tick awareness campaigns) in the face of potential increases in seropositivity in the population.

## Data Availability

The datasets for this manuscript are not publicly available because of data protection regulations. Access to data can, however, be provided to scientists in accordance with the Rhineland Study’s Data Use and Access Policy. Requests to access the datasets should be directed to Dr Monique Breteler, RS-DUAC@dzne.de.

## Abbreviations

*B. burgdorferi* s.l.: *Borrelia burgdorferi* sensu lato
BGS98: German National Health Interview and Examination Survey 1998
DEGS: German Health Interview and Examination Survey for Adults from 2008 to 2011
ELISA: Enzyme-linked immunosorbent assay
IgG: Immunoglobulin G
IgM: Immunoglobulin M
ICH-GCP: International Council for Harmonisation Good Clinical Practice standards
ISCED: International Standard Classification of Education
MIQ 12: Microbiologic-infectiologic quality standard
NRW: North Rhine-Westphalia
VU: Virotech units
SES: Socioeconomic status
